# Supplementary material for: STI1 domain engages transient helices to mediate Dsk2 phase separation and proteasome condensation
Source: EMBO J. 2026 Feb 11;45(8):2712–38. doi: 10.1038/s44318-026-00696-1 (PMC13083955; doi:10.1038/s44318-026-00696-1)
Supplement: Supplementary file 1 — Appendix [file 44318_2026_696_MOESM1_ESM.pdf]

Appendix for

STI1 domain engages transient helices to mediate Dsk2 phase separation and proteasome condensation

Table of Contents

		Page
Appendix Figure S1	Structural (and/or domain) similarities in Dsk2 and human UBQLN2	2
Appendix Figure S2	SDS-PAGE gel of Dsk2 constructs used in this study	4
Appendix Figure S3	Dsk2 predominantly exists in a monomeric state	5
Appendix Figure S4	Amide-amide NOEs reveal helical regions within Dsk2 IDRs	6
Appendix Figure S5	^{1}H-^{15}N TROSY-HSQC spectra of Dsk2 and ΔSTI1 constructs	8
Appendix Figure S6	Probabilities of the interactions between the disordered region residues and STI1 groove from CALVADOS simulations	9
Appendix Figure S7	Internal Dsk2 ALFA tag has no significant impact on Dsk2's function in proteasome condensate formation	10
Appendix Figure S8	Full western blots for determination of K48-linked polyUb and Rpn1-GFP levels in stressed yeast cells	11
Appendix Figure S9	Full western blots for determination of Dsk2ALFA levels in stressed yeast cells	12
Appendix Figure S10	Comparison of NMR relaxation properties for Dsk2 Δ3TH and Dsk2 FL	13
Appendix Table S1	Amino acid sequence of purified Dsk2 constructs	14
Appendix Table S2	SAXS data collection	16
Appendix Table S3	Helix parameters for the three transient helices of Dsk2	18
Appendix Table S4	Molecular weights and molar extinction coefficients of purified Dsk2 constructs used for concentration determination	19
Appendix Table S5	Yeast strain list	20
Appendix Table S6	Plasmids and repair DNAs used for generating different yeast strains	21

A

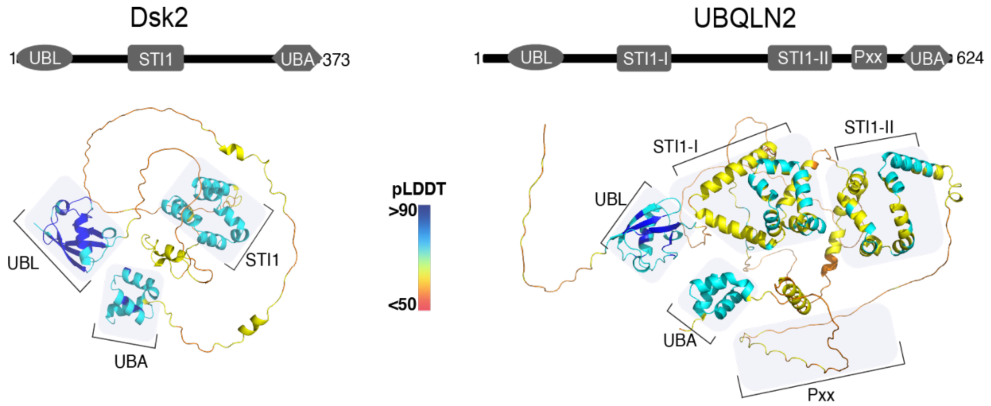

B

```
                                                                          UBL
Dsk2         1 MS-----------------------------------LNIHIKSGQDKWEVNVAPESTVLQFKEAINKANGIPVANQRLIYSGKILKDDQTVE   58
UBQLN1       1 MAES-GESGGPPGSQDSAAGAEGAGAPAAAASAEPKIMKVTVKTPKEKEEFAVPENSSVQQFKEEISKRFKSHTDQLVLIFAGKILKDQDTLS   92
UBQLN2       1 MAEN-GESSGPPRPSRGPAAAQGS----AAAPAEPKIIKVTVKTPKEKEEFAVPENSSVQQFKEAISKRFKSQTDQLVLIFAGKILKDQDTLI   88
UBQLN4       1 MAEPSGAETRPP-------------------------IRVTVKTPKDKEEIVICDRASVKEFKEEISRRFKAQQDQLVLIFAGKILKDGDTLN   68
consensus      *:                                   :.: :*: ::* *. :   .::* :*** *.:        :  **::****** :*:

                 UBL
Dsk2        59 SYHIQDGHSVHLVK-SQPKPQTASA---------AGANNATATGAAAGTGATPNMSSG-QSAGF--NP-------------------------  113
UBQLN1      93 QHGIHDGLTVHLVIKTQNRPQDHSA---------QQT---NTAGSNVTTSSTPNSNSTSGSATSNPFG-------------------------  148
UBQLN2      89 QHGIHDGLTVHLVIKSQNRPQGQST---------QPS---NAAGTNTTSASTPRSNSTPISTNSNPFG-------------------------  144
UBQLN4      69 QHGIKDGLTVHLVIKTPQKAQDPAAATASSPSTPDPA---SAPSTTPASPATPAQPSTSGSASSDAGSGSRRSSGGGPSPGAGEGSPSATASI  158
consensus      .: *:** :****  :  :.*  ::              :   .:..:   : :**    *    *:

                 TH1                                        STI1
Dsk2       114 LADLTSARYAGYLNMPSADMFGPDGGALNND-SNNQDELLRMMENPIFQSQMNEMLSNPQMLDFMIQSNPQLQAMG---PQARQMLQSP----  198
UBQLN1     149 LGGLGGLAGLSSLGLNTTNF-SELQSQMQRQLLSNPEMMVQIMENPFVQS----MLSNPDLMRQLIMANPQMQQLIQRNPEISHMLNNPDIMR  236
UBQLN2     145 LGSLGGLAGLSSLGLSSTNF-SELQSQMQQQLMASPEMMIQIMENPFVQS----MLSNPDLMRQLIMANPQMQQLIQRNPEISHLLNNPDIMR  232
UBQLN4     159 LSGFGGILGLGSLGLGSANF-MELQQQMQRQLMSNPEMLSQIMENPLVQD----MMSNPDLMRHMIMANPQMQQLMERNPEISHMLNNPELMR  246
consensus      *..: .    . *.: ::::          ::.:   . : : ::****:.*.    *:***::  :* :***:* :     *:  ::*:.*

                          STI1                            STI1
Dsk2       199 ----------MFRQMLTN----------------------PDMIRQSMQFARMMDPN---AGMGSAGG-----------AASAFPAPGGDAPE  245
UBQLN1     237 QTLELARNPAMMQEMMRNQDRALSNLESIPGGYNALRRMYTDIQEPMLSAAQEQFGGNPFASLVSNTSSGEGSQPSRTENRDPLPNPWAPQTS  329
UBQLN2     233 QTLEIARNPAMMQEMMRNQDLALSNLESIPGGYNALRRMYTDIQEPMLNAAQEQFGGNPFASVGSSSSSGEGTQPSRTENRDPLPNPWAPPPA  325
UBQLN4     247 QTMELARNPAMMQEMMRNQDRALSNLESIPGGYNALRRMYTDIQEPMFSAAREQFGNNPFSSLAGNSDSS-SSQPLRTENREPLPNPWSPSPP  338
consensus                *:::*: *                       .*: .  :. *:    .   :.: .  .               ..:* * .  .

                                                                                        TH2
Dsk2       246 EGSNTNTTSSSNTGNNAGTNAGTNAGA--N-----------------------------------TAANPFASLLNPALNP------FANAG-  294
UBQLN1     330 QSSSASSGTASTVGGTTGSTASGTSGQS-TTAPNLVPGVGASMFNTPGMQSLLQQITENPQLMQNMLSAPYMRSMMQSLSQNPDLAAQMMLNN  421
UBQLN2     326 TQSSATTSTTTSTGSGSGNSSSNATGNT-VAAANYV----ASIFSTPGMQSLLQQITENPQLIQNMLSAPYMRSMMQSLSQNPDLAAQMMLNS  413
UBQLN4     339 TSQAPGSGGEGTGGSGTSQVHPTVSNPFGINAASLG----SGMFNSPEMQALLQQISENPQLMQNVISAPYMRSMMQTLAQNPDFAAQMMVNV  427
consensus        . . :    . *. :.      :.                                         : *:   :  :*              .

                       TH3
Dsk2       295 ------NAASTGMPAFDPA-----------------------------------------------------------------------  307
UBQLN1     422 PLFAGNPQLQEQMRQQLPTFLQQMQNPDTLSAMSNPRAMQALLQIQQGLQTLATEAPGLIPGFTPG-----L--------------------  488
UBQLN2     414 PLFTANPQLQEQMRPQLPAFLQQMQNPDTLSAMSNPRAMQALMQIQQGLQTLATEAPGLIPSFTPGVGVGVLGTAIGPVGPVTPIGPIGPIVP  506
UBQLN4     428 PLFAGNPQLQEQLRLQLPVFLQQMQNPESLSILTNPRAMQALLQIQQGLQTLQTEAPGLVPSL------------------------------  490
consensus                .  :    *.

                                                                       TH3                UBA
Dsk2       308 ------------------------------------------------------LLASMFQPPVQASQAEDTRPPEERYEHQLRQLNDMGFF  345
UBQLN1     489 -------GALGST-G-----------GSSGTNGSNATPSENTSPTAGTTEPGHQQFIQQMLQALA-GV-NPQLQNPEVRFQQQLEQLSAMGFL  560
UBQLN2     507 FTPIGPIGPIGPT-GPAAPPGSTGSGGPTGPTVSSAAPSETTSPTSESG--PNQQFIQQMVQALA-GANAPQLPNPEVRFQQQLEQLNAMGFL  595
UBQLN4     491 -------GSFGISRTPAPSAGSNAGSTPEAPTSSPATPA-TSSPTGASS--AQQQLMQQMIQLLA-GSGNSQVQTPEVRFQQQLEQLNSMGFI  572
consensus                                                              :: .*.*  . .    :   ** *:::**.**. ***:

                   UBA
Dsk2       346 DFDRNVAALRRSGGSVQGALDSLLNGD-V  373
UBQLN1     561 NREANLQALIATGGDINAAIERLLGSQPS  589
UBQLN2     596 NREANLQALIATGGDINAAIERLLGSQPS  624
UBQLN4     573 NREANLQALIATGGDINAAIERLLGSQLS  601
consensus      : : *: **  :**.::.*:: **..:
```

Appendix Figure S1. Structural (and/or domain) similarities in Dsk2 and human UBQLN2.
(A) AlphaFold predicted structures of Dsk2 and UBQLN2 are compared along with their

respective domain architectures. Individual domains have been labeled. Protein secondary structures have been colored according to their prediction confidence score (pLDDT score). (B) A multiple sequence alignment generated with M-Coffee (Moretti *et al*, 2007; Wallace *et al*, 2006; Di Tommaso *et al*, 2011) reveals conserved regions between yeast Dsk2 and human UBQLNs (1, 2, and 4). The UBL domain, UBA domain, and part of the STI1 domain of Dsk2 show higher consensus/conservation with human UBQLNs compared to the rest of the protein. (conserved (*), conservative substitution (:), semi-conservative substitution (.))

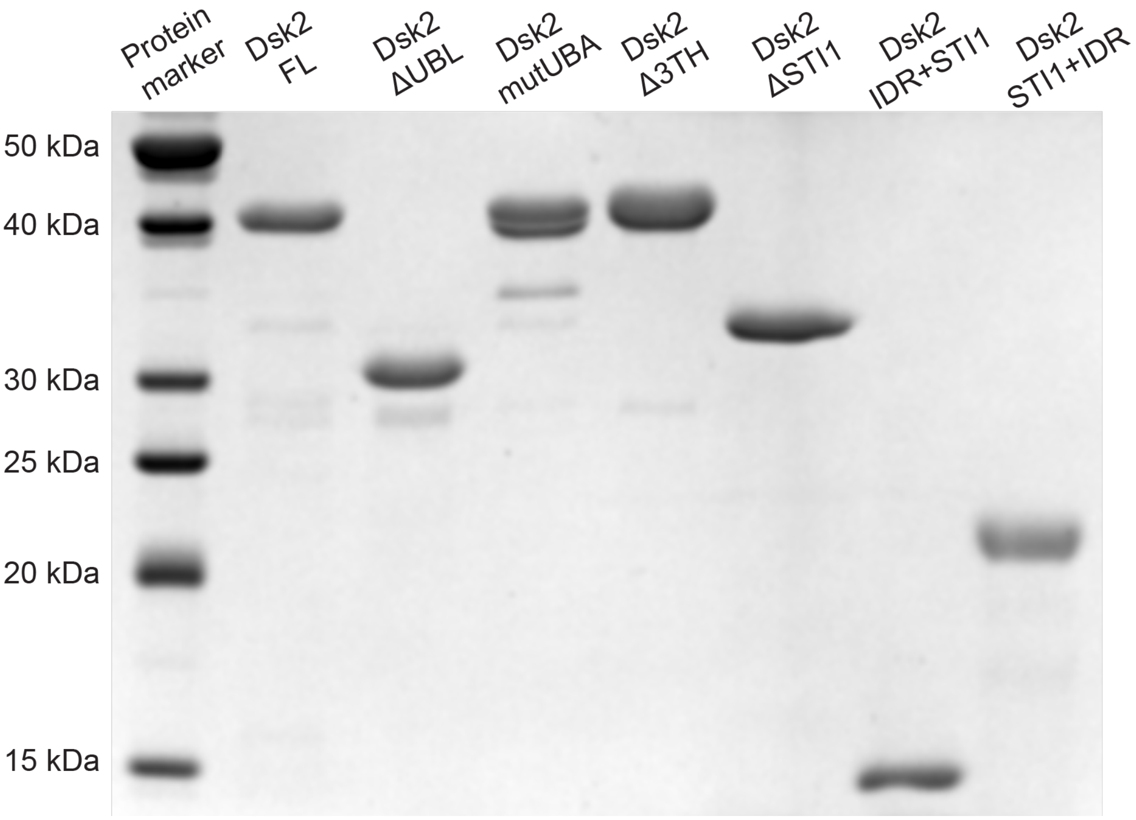

Appendix Figure S2. SDS-PAGE gel of Dsk2 constructs used in this study. SDS-PAGE analysis shows that all Dsk2 constructs (Dsk2 FL, Dsk2 ΔUBL, Dsk2 mutUBA, Dsk2 Δ3TH, Dsk2 ΔSTI1, Dsk2 IDR+STI1, Dsk2 STI1+IDR) were successfully expressed and purified with high purity (>90%).

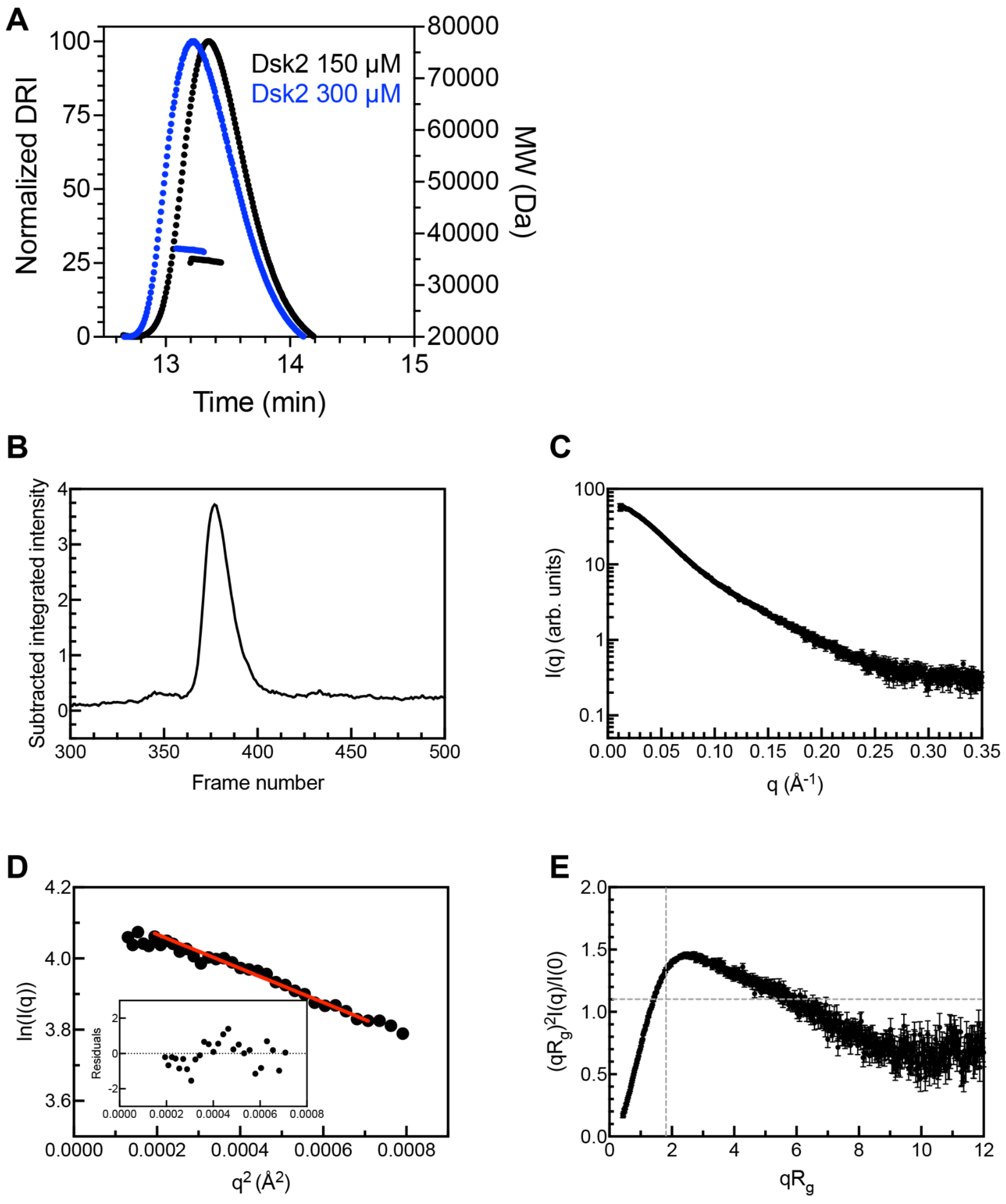

Appendix Figure S3. Dsk2 predominantly exists in a monomeric state. (A) SEC-MALS analysis shows that Dsk2 is a monomer at 150 µM (black) and 300 µM (blue) protein concentration using NMR buffer at pH 6.8. A slightly earlier elution at the higher concentration suggests formation of dynamic higher order self-assemblies/association. (B) SEC-SAXS profiles for Dsk2 at 150 µM. (C) I(q) vs. q scattering curve determined from frames 378-389 on the corresponding SEC-SAXS profiles in B. (D) Guinier plot with red line showing the linear fit of ln(I(q)) vs. q^2, while inset shows residuals of fit. (E) Dimensionless Kratky plot includes dashed lines to indicate where a globular protein would peak.

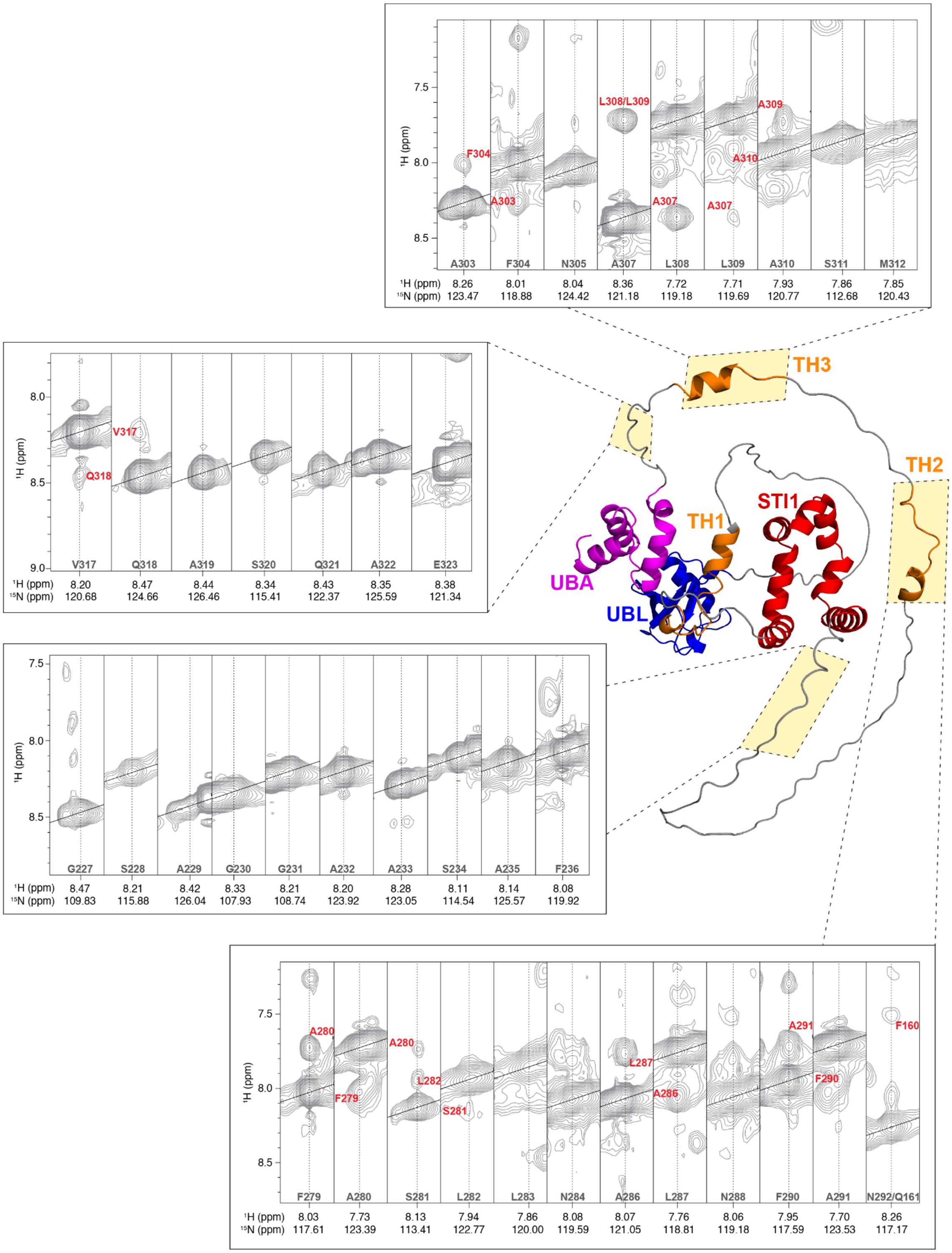

Appendix Figure S4. Amide-amide NOEs reveal helical regions within Dsk2 IDRs. Strip plots from the 3D ^{1}H-^{15}N HSQC-NOESY spectra are shown for selected regions of Dsk2 IDRs (see AlphaFold-predicted structure of Dsk2), highlighting amide-amide NOE cross-peaks. Each strip corresponds to a specific ^{15}N chemical shift, with the amide ^{1}H-^{1}H peaks displayed along

the horizontal axis. Within a strip, the self peak is indicated by intersection of the solid black diagonal line and a dashed vertical line. Corresponding residue (i) is labeled below each strip. Amide–amide NOE cross-peaks (inter-residue NOEs) are marked in red at their respective positions, indicating spatial proximity between backbone amide protons wherever shown. We observed strong i±1 amide-amide NOEs within the TH2 and TH3 regions supporting the presence of helical secondary structure, while disordered regions (middle two strip plots) exhibit very weak or no amide-amide NOEs. To reduce spectral complexity, we used the STI1+IDR construct (contains TH2 and TH3) for this HSQC-NOESY experiment.

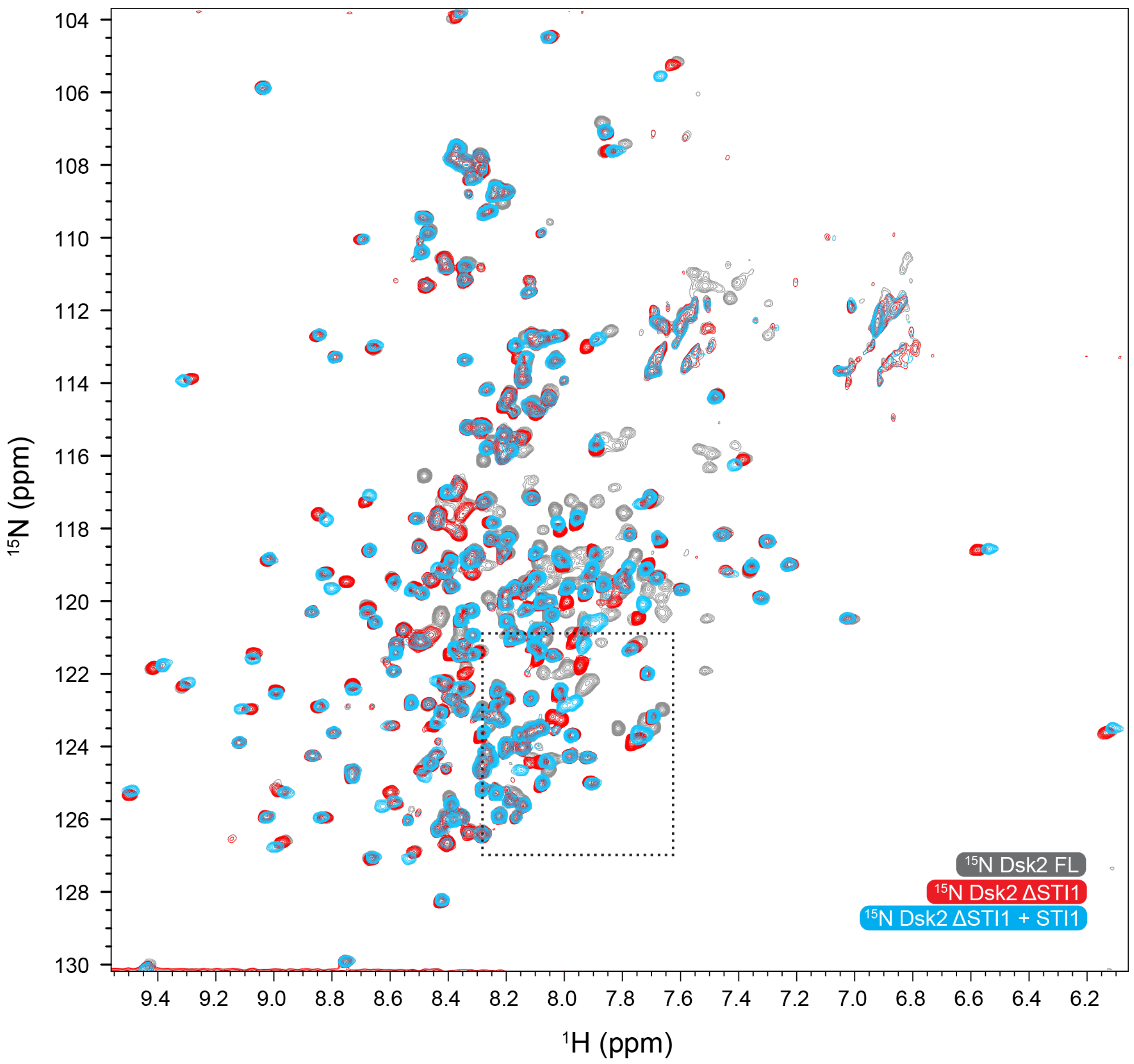

Appendix Figure S5. ^{1}H-^{15}N TROSY-HSQC spectra of Dsk2 and ΔSTI1 constructs. Full spectra correspond to the zoomed-in region (dashed box) shown in Figure 4A with peaks color-coded according to legend. All spectra acquisition parameters (number of scans, receiver gain, etc.) were identical.

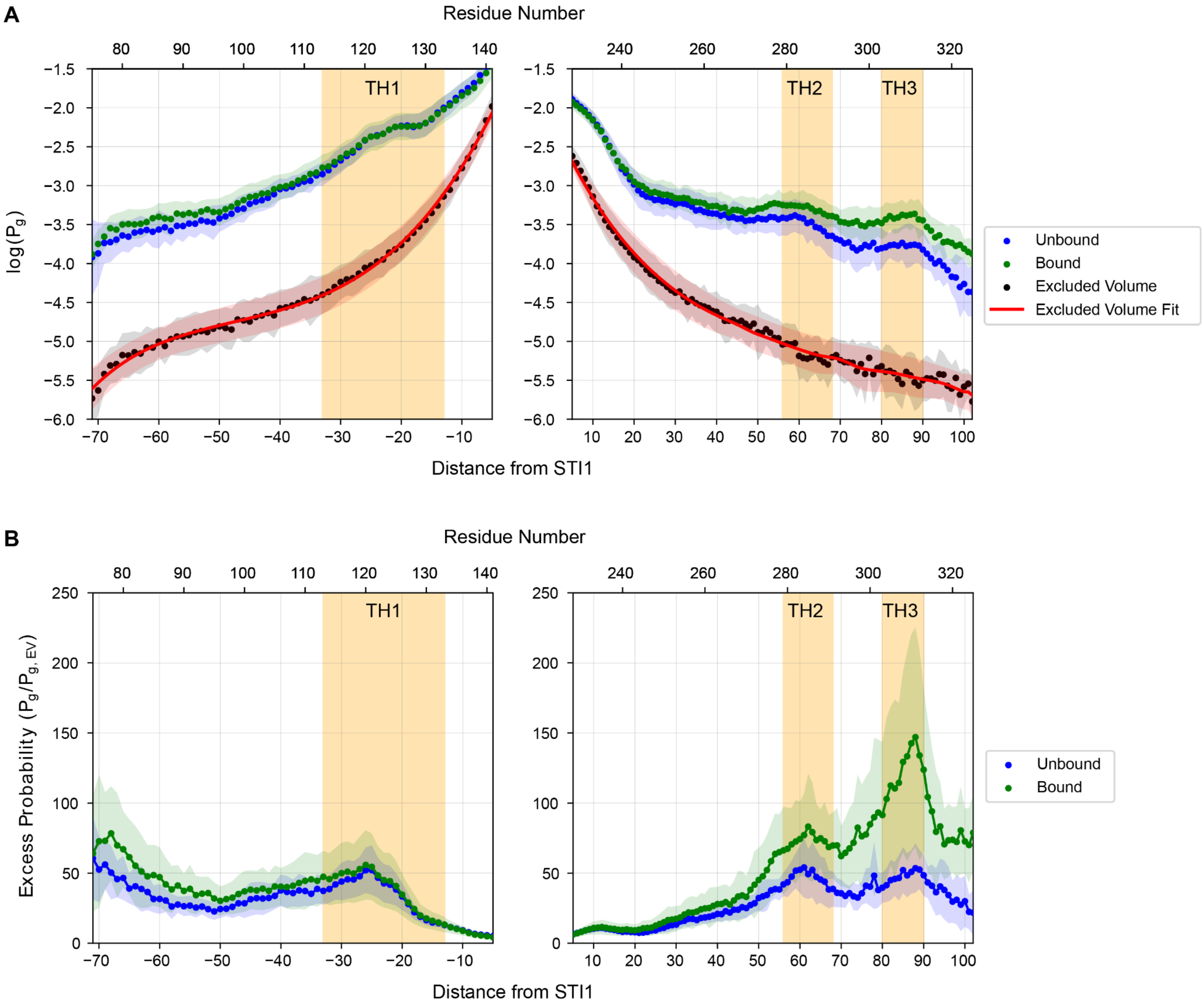

Appendix Figure S6. Probabilities of the interactions between the disordered region residues and STI1 groove from CALVADOS simulations. (A) Log of probability of the disordered region residues occupying the STI1 groove where the UBL and UBA were unbound (blue) or bound (green) compared to excluded volume (EV) simulations where attractions were turned off (black). Shaded colors represent standard deviation among ten replicates that use different initial conformations. EV simulations (see Methods) establish a baseline occupancy of the STI1 domain based on steric proximity alone and were fitted with a spline (red). TH regions are colored with an orange background. (B) Excess probability, calculated as the probability ratio between the regular and EV simulations, for UBL and UBA unbound (blue) and bound (green). Values greater than 1 indicate enrichment beyond sterics, consistent with sequence-specific interactions.

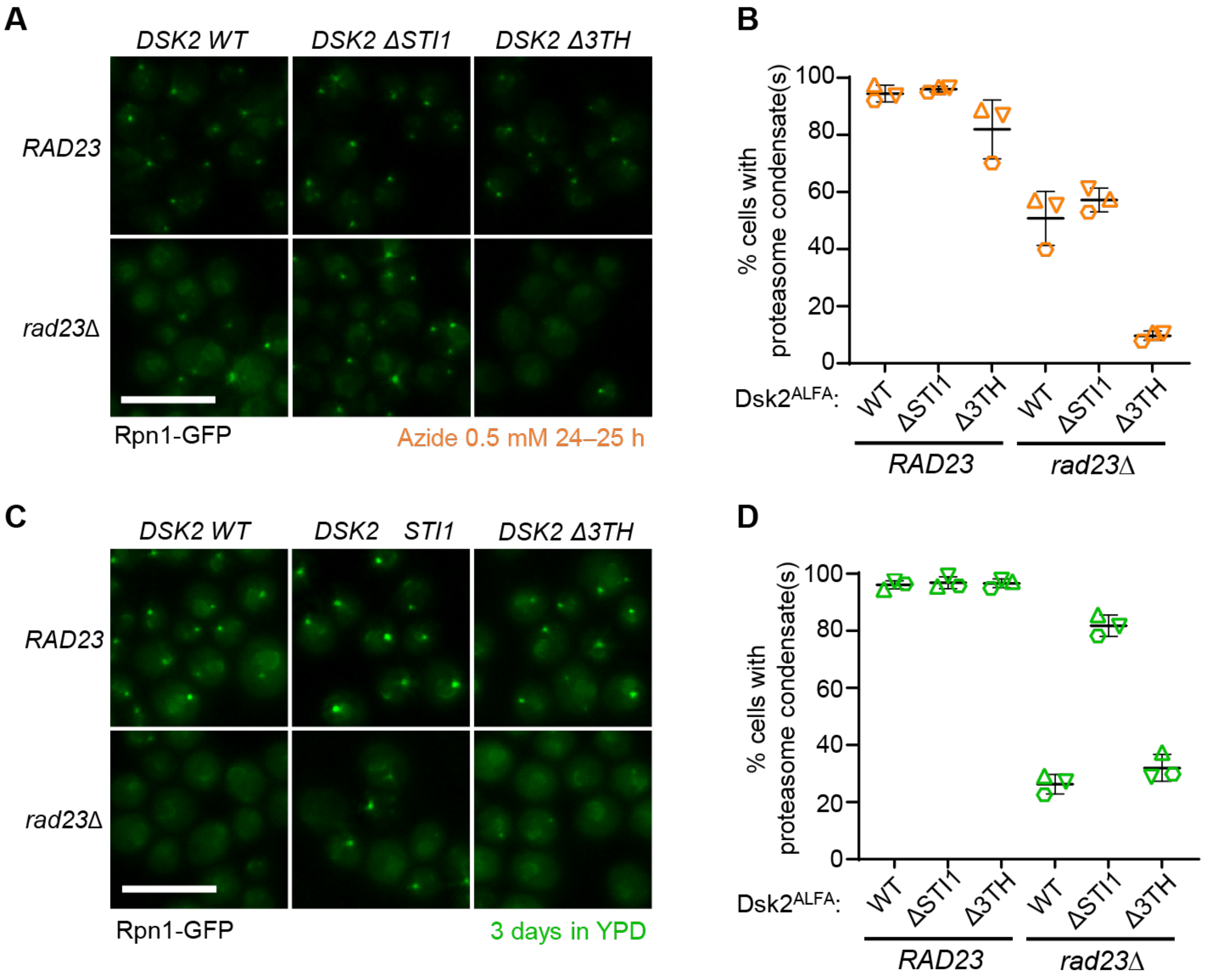

Appendix Figure S7. Internal Dsk2 ALFA tag has no significant impact on Dsk2's function in proteasome condensate formation. To validate the ALFA tag (inserted between Dsk2 Glu246 and Gly247) with regard to proteasome condensate formation, cells were treated and analyzed as in strains with untagged Dsk2 in Figure 6 and Figure EV2. (A) Extended depth-of-field epifluorescence images of the GFP channel (Rpn1-GFP) representative of three biological replicates in RAD23 and *rad23Δ* cells treated with azide for 24 to 25 hours. (B) Quantification of the percent of cells with at least one proteasome condensate. Compare to Figure 6B. (C and D) Similar experiments as shown in A and B, respectively, for cells under prolonged growth stress. Compare to Figure 6H. On plots, horizontal line and error bars represent mean and SD, respectively.

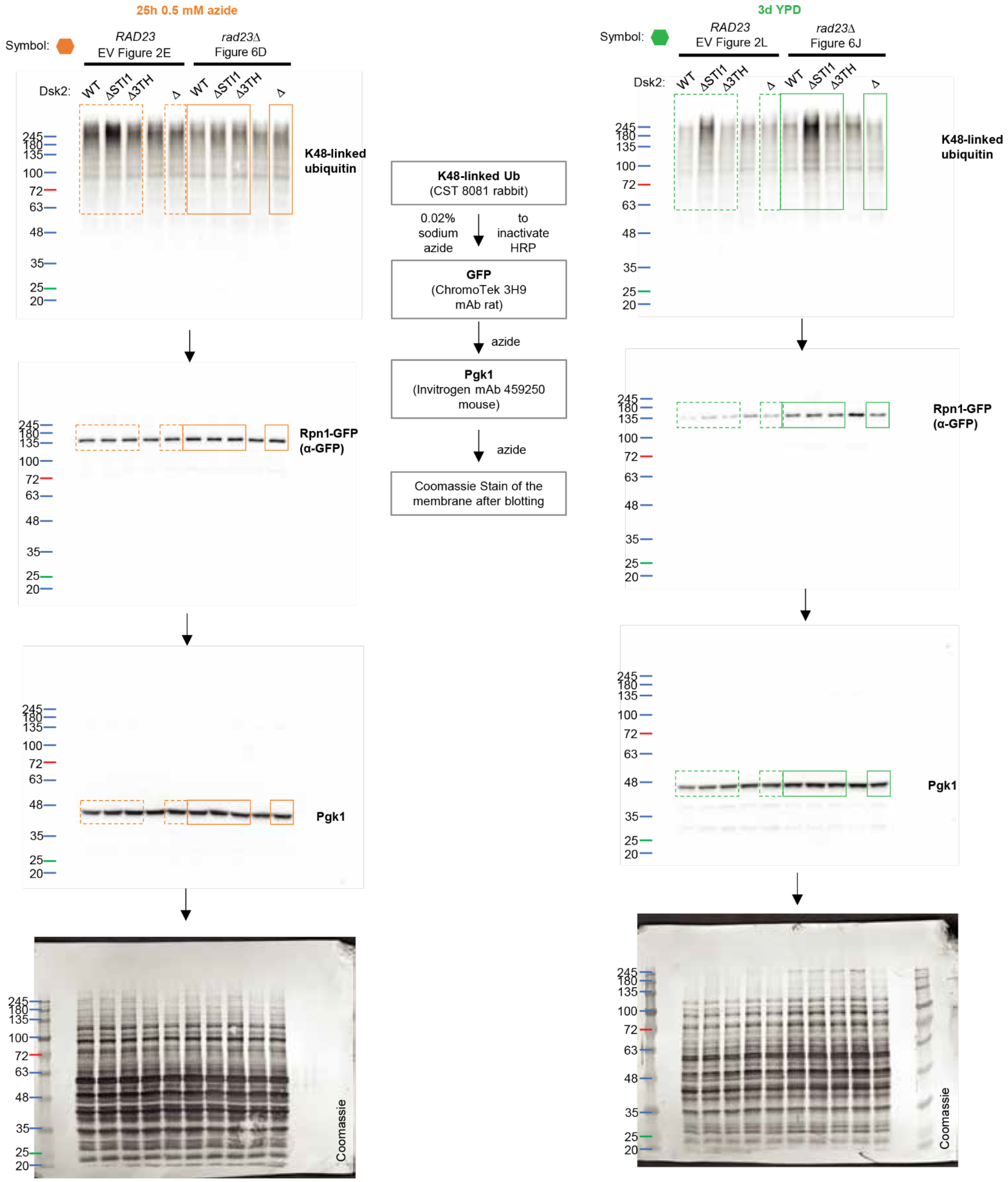

Appendix Figure S8. Full western blots for determination of K48-linked polyUb and Rpn1-GFP levels in stressed yeast cells. Full membranes of the western blots depicted in Figure 6D, 6J and Figure EV2E, EV2L, under azide and prolonged growth (3d YPD) stress conditions. Highlighted boxes indicate regions displayed in the main and EV figures (as mentioned on the top). Membranes were sequentially reprobed with primary antibodies from different species, using 0.02% sodium azide in TBST to inactivate horseradish peroxidase-conjugated secondary antibodies between probings, as outlined in the flowchart. Orange and green hexagon symbols denote representative experiments and correspond to those shown in the graphs of Figure 6 and Figure EV2.

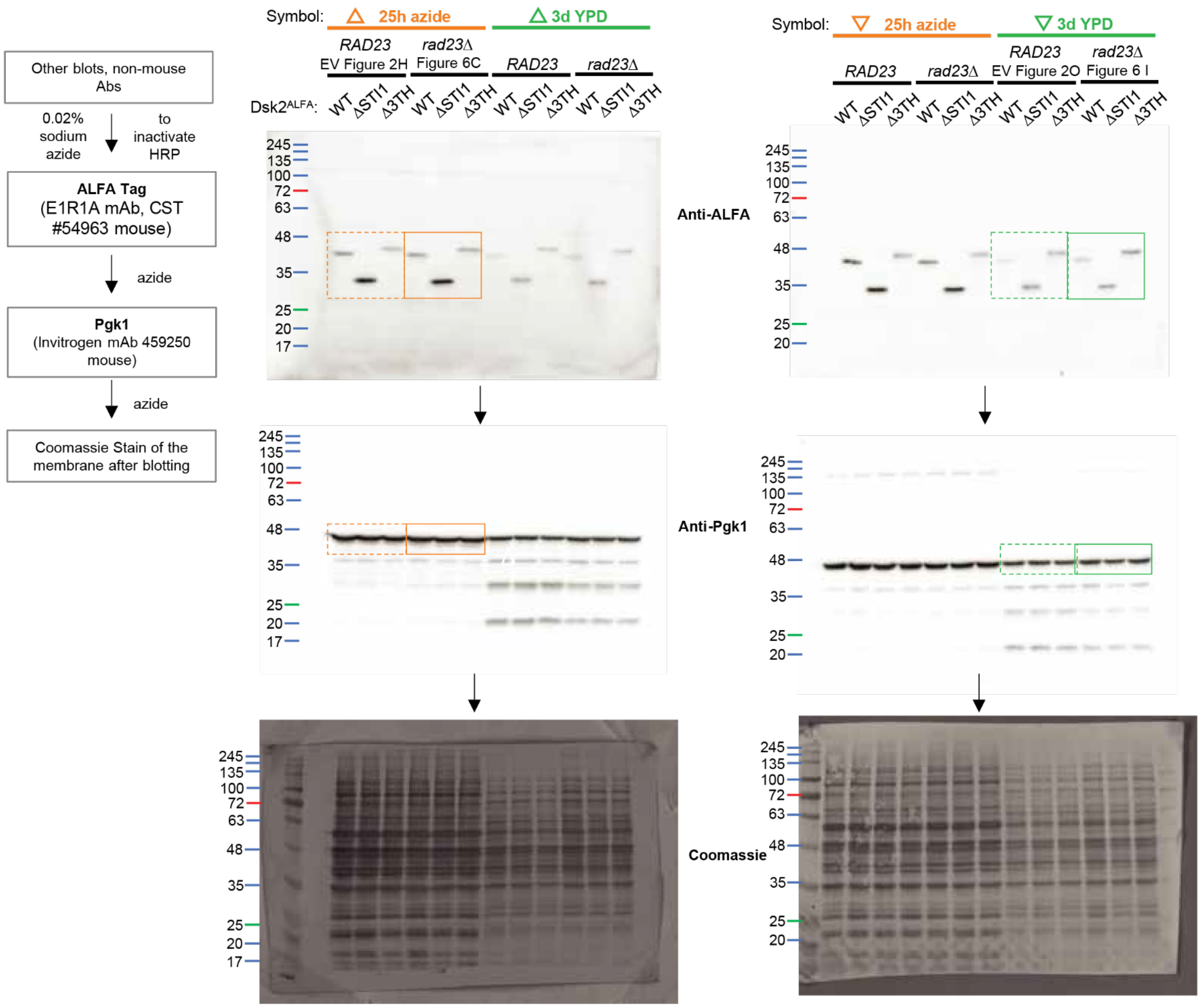

Appendix Figure S9. Full western blots for determination of Dsk2^{ALFA} levels in stressed yeast cells. Full membranes of the western blots depicted in Figure 6C, 6I and Figure EV2H, EV2O, under azide and prolonged growth (3d YPD) stress conditions. Highlighted boxes indicate regions displayed in the main and EV figures (as mentioned on the top). Membranes were sequentially reprobed with primary antibodies from different species, using 0.02% sodium azide in TBST to inactivate horseradish peroxidase-conjugated secondary antibodies between probings, as outlined in the flowchart. Orange and green triangle symbols denote representative experiments and correspond to those shown in the graphs of Figure 6 and Figure EV2.

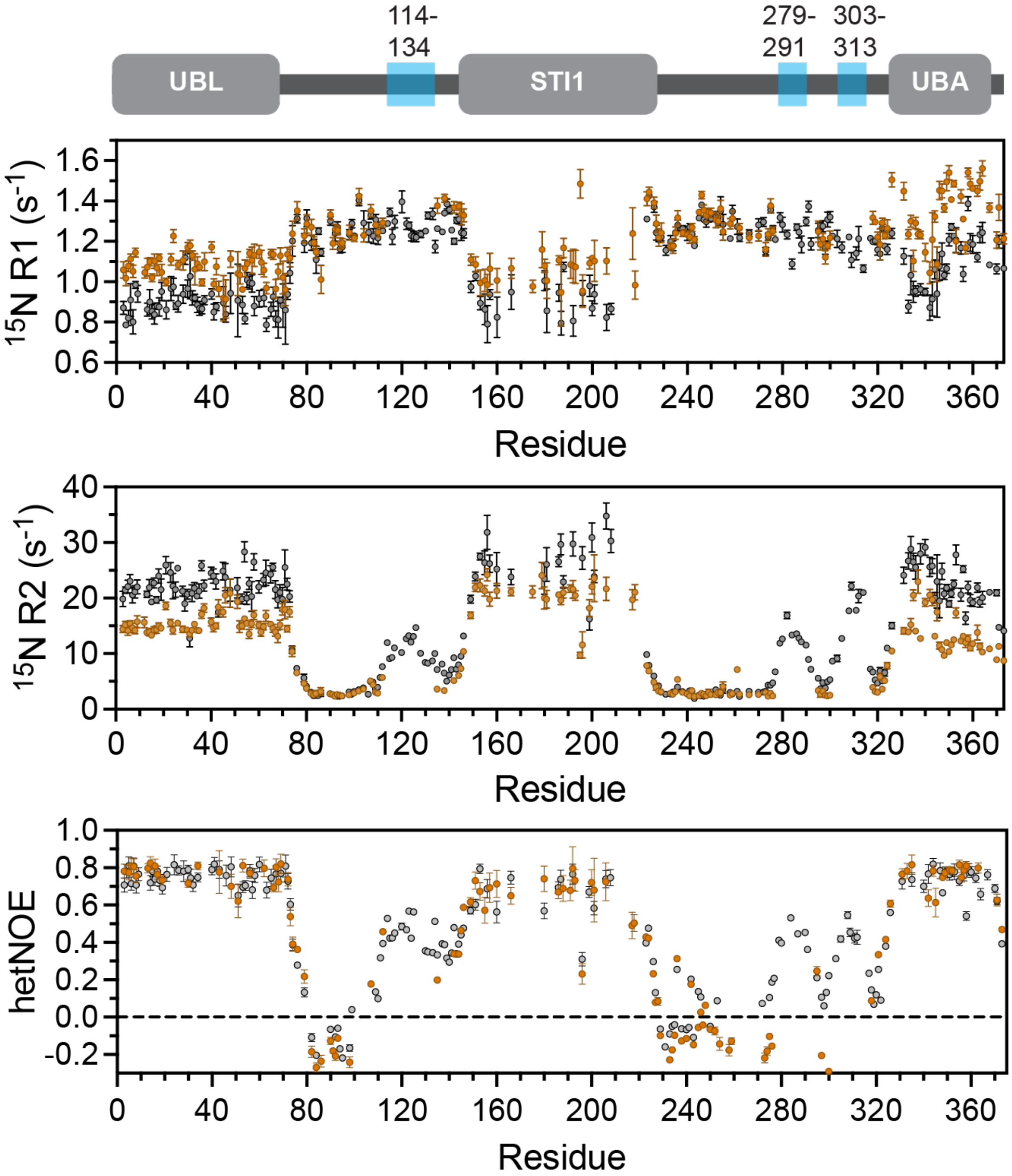

Appendix Figure S10. Comparison of NMR relaxation properties for Dsk2 Δ3TH and Dsk2 FL. Comparison of ^{15}N R_1 and R_2 relaxation rates, and hetNOE values between Dsk2 FL (gray) and Dsk2 Δ3TH (brown). Errors in R_1 and R_2 relaxation rates were determined using 500 Monte Carlo trials using RELAXFIT. Errors in hetNOE measurements were determined using the SE propagation formula. The increase in R_1 relaxation rates and corresponding decrease in R_2 relaxation rates of Dsk2 Δ3TH in all folded domains (UBL, STI1, and UBA) indicate loosening of structural compactness (has become more flexible) compared to the Dsk2 FL. A comparison of the hetNOE values reveals that deletion of all three transient helical regions has no significant effect on the fast local dynamics throughout the protein.

Appendix Table S1. Amino acid sequence of purified Dsk2 constructs. Color coding: UBL (blue), transient helical regions (orange), STI1 domain (red), and UBA (violet).

Construct	Amino acid sequence
Dsk2 FL	MSLNIHIKSGQDKWEVNVAPESTVLQFKEAINKANGIPVANQRLIYSGKILKDDQTVESYHIQDGHSVHLVKSQPKPQTASAAGANNATATGAAAGTGATPNMSSGQSAGFNPLADLTSARYAGYLNMPSADMFGPDGGALNNDSNNQDELLRMMENPIFQSQMNEMLSNPQMLDFMIQSNPQLQAMGPQARQMLQSPMFRQMLTNPDMIRQSMQFARMMDPNAGMGSAGGAASAFPAPGGDAPEEGSNTNTTSSSNTGNNAGTNAGTNAGANTAANPFASLLNPALNPFANAGNAASTGMPAFDPALLASMFQPPVQASQAEDTRPPEERYEHQLRQLNDMGFFDFDRNVAALRRSGGSVQGALDSLLNGDV
Dsk2 ΔUBL	KPQTASAAGANNATATGAAAGTGATPNMSSGQSAGFNPLADLTSARYAGYLNMPSADMFGPDGGALNNDSNNQDELLRMMENPIFQSQMNEMLSNPQMLDFMIQSNPQLQAMGPQARQMLQSPMFRQMLTNPDMIRQSMQFARMMDPNAGMGSAGGAASAFPAPGGDAPEEGSNTNTTSSSNTGNNAGTNAGTNAGANTAANPFASLLNPALNPFANAGNAASTGMPAFDPALLASMFQPPVQASQAEDTRPPEERYEHQLRQLNDMGFFDFDRNVAALRRSGGSVQGALDSLLNGDV
Dsk2 mutUBA	MSLNIHIKSGQDKWEVNVAPESTVLQFKEAINKANGIPVANQRLIYSGKILKDDQTVESYHIQDGHSVHLVKSQPKPQTASAAGANNATATGAAAGTGATPNMSSGQSAGFNPLADLTSARYAGYLNMPSADMFGPDGGALNNDSNNQDELLRMMENPIFQSQMNEMLSNPQMLDFMIQSNPQLQAMGPQARQMLQSPMFRQMLTNPDMIRQSMQFARMMDPNAGMGSAGGAASAFPAPGGDAPEEGSNTNTTSSSNTGNNAGTNAGTNAGANTAANPFASLLNPALNPFANAGNAASTGMPAFDPALLASMFQPPVQASQAEDTRPPEERYEHQLRQLNDMAAFDFDRNVAALRRSGGSVQGALDSLLNGDV
Dsk2 Δ3TH	MSLNIHIKSGQDKWEVNVAPESTVLQFKEAINKANGIPVANQRLIYSGKILKDDQTVESYHIQDGHSVHLVKSQPKPQTASAAGANNATATGAAAGTGATPNMSSGQSAGFNPGPDGGALNNDSNNQDELLRMMENPIFQSQMNEMLSNPQMLDFMIQSNPQLQAMGPQARQMLQSPMFRQMLTNPDMIRQSMQFARMMDPNAGMGSAGGAASAFPAPGGDAPEEGSNTNTTSSSNTGNNAGTNAGTNAGANTAANPNAGNAASTGPPVQASQAEDTRPPEERYEHQLRQLNDMGFFDFDRNVAALRRSGGSVQGALDSLLNGDV
Dsk2 ΔSTI1	MSLNIHIKSGQDKWEVNVAPESTVLQFKEAINKANGIPVANQRLIYSGKILKDDQTVESYHIQDGHSVHLVKSQPKPQTASAAGANNATATGAAAGTGATPNMSSGQSAGFNPLADLTSARYAGYLNMPSADMFGPDGGALNNDAGMGSAGGAASAFPAPGGDAPEEGSNTNTTSSSNTGNNAGTNAGTNAGANTAANPFASLLNPALNPFANAGNAASTGMPAFDPALLASMFQPPVQASQAEDTRPPEERYEHQLRQLNDMGFFDFDRNVAALRRSGGSVQGALDSLLNGDV

Dsk2 IDR+STI1	KPQTASAAGANNATATGAAAGTGATPNMSSGQSAGFNPLADLTSARYAGYLNMPSADMFGPDGGALNNDSNNQDELLRMMENPIFQSQMNEMLSNPQMLDFMIQSNPQLQAMGPQARQMLQSPMFRQMLTNPDMIRQSMQFARMMDPN
Dsk2 STI1+IDR	SNNQDELLRMMENPIFQSQMNEMLSNPQMLDFMIQSNPQLQAMGPQARQMLQSPMFRQMLTNPDMIRQSMQFARMMDPNAGMGSAGGAASAFPAPGGDAPEEGSNTNTTSSSNTGNNAGTNAGTNAGANTAANPFASLLNPALNPFANAGNAASTGMPAFDPALLASMFQPPVQASQAEDTR
Dsk2 UBL	MSLNIHIKSGQDKWEVNVAPESTVLQFKEAINKANGIPVANQRLIYSGKILKDDQTVESYHIQDGHSVHLVKSQP
Dsk2 UBA	RPPEERYEHQLRQLNDMGFFDFDRNVAALRRSGGSVQGALDSLLNGDV

Appendix Table S2. SAXS data collection

(a) Sample details	
Organism	*S. cerevisiae*
Source (Catalogue No. or reference)	Expressed in *E. coli* (this work)
Description: sequence (including Uniprot ID + uncleaved tags), bound ligands/modifications, etc.	Full-length Dsk2 (no tags), Uniprot ID P48510
Extinction coefficient ε in M^{-1} cm^{-1} (wavelength in nm)	12950 (280)
Molecular mass M from chemical composition (Da)	39,345
For SEC-SAS, loading volume/concentration (mg ml^{-1}), injection volume (μl), flow rate (ml min^{-1})	6.11, 300, 0.65
Solvent composition and source	pH 6.8 20 mM NaPhos, 0.5 mM EDTA, 0.02% NaN_3
(b) SAS data collection parameters	
Instrument	SIBYLS facility (beamline 12.3.1) at the Advanced Light Source, with Pilatus X3 2M detector (Dectris)
Wavelength (Å)	1.240
Camera length (m)	2.077
q-measurement range	0.0114-0.0473
Normalization	Transmitted intensity
Exposure time/number	2.0 seconds (600 frames)

Sample Configuration	SEC-MALS-SAXS using a Shodex KW-803 and an Agilent 1260 Series HPLC. UV data was measured with an Agilent 1290 DAD, and MALS/RI data by DAWN HELEOS-II (18-angle) and Optilab T-rEX (RI) instruments (Wyatt Technology). SAXS data was measured in a 1 mm, Mica-windowed flow cell
Sample Temperature	25 ^{o}C
(c) Software employed	
SAXS data reduction	Radial averaging; frame comparison, averaging, and subtraction done using BioXTAS RAW 2.1.1 (Hopkins *et al*, 2017)
Basic analysis: Guinier, M.W. P(r)	Guinier fit and M.W. using BioXTAS RAW. RAW uses MoW and Vc M.W. methods (Rambo & Tainer, 2013; Piiadov *et al*, 2019)
MALS-RI analysis	Astra 7 (Wyatt)
(d) Structural Parameters	
Guinier Analysis	Full-length Dsk2
I(0)	64.32 ± 0.65
R_g (Å)	37.90 ± 0.73
q-range (Å^{-1})	0.01394 – 0.02661
Quality-of-fit parameter (with definition)	0.9706 (r^2)
M from MALS (kDa)	35

Appendix Table S3. Helix parameters for the three transient helices of Dsk2. All hydrophobic amino acids (in the order of increasing hydrophobicity: A, M, L, and F) have been highlighted in red in each sequence, showing their number and distribution in respective helices.

Transient helix	Helix length	Sequence	Number of hydrophobic amino acids
TH1 (114-126)	13	**LA**D**L**TS**A**RY**A**GY**L**	6
TH2 (279-291)	13	**FA**S**LL**NP**AL**NP**FA**	8
TH3 (303-314)	12	**AF**DP**ALLA**S**MF**Q	8

Appendix Table S4. Molecular weights and molar extinction coefficients of purified Dsk2 constructs used for concentration determination.

**Construct**	**Molecular weight (Da)**	**Molar extinction coefficient ($M^{-1}cm^{-1}$)**
Dsk2 FL	39345	12950
Dsk2 ΔUBL	30969	4470
Dsk2 mutUBA	39283	12950
Dsk2 Δ3TH	34178	9970
Dsk2 ΔSTI1	30007	12950
Dsk2 IDR+STI1	15945	2980
Dsk2 STI1+IDR	19106	NA*

NA*: Not applicable*
**Concentration of Dsk2 STI1+IDR was estimated by SDS-PAGE gel using concentration standards of a similar molecular weight protein.*

Appendix Table S5. Yeast strain list

Strain	Simplified name	Genes manipulated*	Figures	Ref
sJR1127[A]	*rad23Δ DSK2 WT*	*rpn1*::*RPN1-GFP* (HIS3) *rad23Δ*::KanMX	6	a
sJR2660[A]	*rad23Δ DSK2 ΔSTI1*	*rpn1*::*RPN1-GFP* (HIS3) *rad23Δ*::KanMX *dsk2*::*DSK2-S145–N223del*	6	b
sJR2690[A]	*rad23Δ DSK2 Δ3TH*	*rpn1*::*RPN1-GFP* (HIS3) *rad23Δ*::KanMX *dsk2*::*DSK2-L114–F134del, F279–A291del, M301–Q314del*	6	b
sJR1203[A]	*rad23Δ dsk2Δ*	*rpn1::RPN1-GFP (HIS3) rad23Δ::KanMX dsk2Δ::natMX4*	6	a
sJR2692[A]	*rad23Δ DSK2^{ALFA} WT*	*rpn1*::*RPN1-GFP* (HIS3) *rad23Δ*::KanMX *dsk2*::*DSK2-E246_G247insALFA*	6, S7	b
sJR2747[A]	*rad23Δ DSK2^{ALFA} ΔSTI1*	*rpn1*::*RPN1-GFP* (HIS3) *rad23Δ*::KanMX *dsk2*::*DSK2-S145–N223del, E246_G247insALFA*	6, S7	b
sJR2749[A]	*rad23Δ DSK2^{ALFA} Δ3TH*	*rpn1*::*RPN1-GFP* (HIS3) *rad23Δ*::KanMX *dsk2*::*DSK2-L114–F134del, E246-_G247insALFA, F279–A291del, M301–Q314del*	6, S7	b
sJR1255[B]	*DSK2 WT*	*rpn1*::*RPN1-GFP* (HIS3)	EV2	a
sJR2659[B]	*DSK2 ΔSTI1*	*rpn1*::*RPN1-GFP* (HIS3) *dsk2*::*DSK2-S145–N223del*	EV2	b
sJR2689[B]	*DSK2 Δ3TH*	*rpn1*::*RPN1-GFP* (HIS3) *dsk2*::*DSK2-L114–F134del, F279–A291del, M301–Q314del*	EV2	b
sJR1123[B]	*dsk2Δ*	*rpn1::RPN1-GFP (HIS3) dsk2Δ::KanMX*	EV2	a
sJR2691[B]	*DSK2^{ALFA} WT*	*rpn1*::*RPN1-GFP* (HIS3) *dsk2*::*DSK2-E246_G247insALFA*	EV2, S7	b
sJR2746[B]	*DSK2^{ALFA} ΔSTI1*	*rpn1*::*RPN1-GFP* (HIS3) *dsk2*::*DSK2-S145–N223del, E246_G247insALFA*	EV2, S7	b
sJR2748[B]	*DSK2^{ALFA} Δ3TH*	*rpn1*::*RPN1-GFP* (HIS3) *dsk2*::*DSK2-L114–F134del, E246_G247insALFA, F279–A291del, M301–Q314del*	EV2, S7	b
sJR1323[B]	*α1-mCherry Dsk2-GFP*	*scl1::SCL1-mCherry* (HYG) *dsk2::DSK2-GFP* (HIS3)	EV2	b
sJR2662[B]	*α1-mCherry Dsk2 ΔSTI1-GFP*	*scl1::SCL1-mCherry* (HYG) *dsk2::DSK2-S145–N223del-GFP* (HIS3)	EV2	b

* Genotype nomenclature for CRISPR-generated alleles was adapted from (Mucelli & Huang, 2024) to indicate deletions including the specified wild-type codons using an en dash (e.g. *S145–N223del)* and insertion of the ALFA Tag between the specified wild-type codons using an underscore (e.g. *E246_G247insALFA*).

Strain backgrounds

A. MATα his3*Δ*1 leu2*Δ*0 lys2*Δ*0 ura3*Δ*0 (BY4742)

B. Haploid dissected from BY4743

References: a. (Waite *et al*, 2024), b. This study

Appendix Table S6. Plasmids and repair DNAs used for generating different yeast strains

**Plasmids**		
**ID**	**Purpose**	**Sequence**
pJR1173[a]	Cas9/sgRNA plasmid used with the pRL1528 repair DNA to delete codons corresponding to Dsk2 STI1 domain (aa145–223)	5'-TGTAGCATTTGCCTGGCTTGGTTTTAGAGCTAG-3'
pJR1189[a]	Cas9/sgRNA plasmid used with the pRL1573 repair DNA to delete codons corresponding to Dsk2 TH2 (aa279–291) and TH3 (aa301–314)	5'-CGTTTCCCGCGTTAGCAAAGGTTTTAGAGCTAG-3'
pJR1194[a]	Cas9/sgRNA plasmid used with the pRL1574 repair DNA to delete codons corresponding to Dsk2 TH1 (aa114–134)	5'-GCACTGGTCAAGTCGGCCAGGTTTTAGAGCTAG-3'
pJR1201[a]	Cas9/sgRNA plasmid used with the pRL1551 repair DNA to insert the ALFA Tag between E246 and G247 of Dsk2	5'-TTCGTGTTGGAGCCTTCCTCGTTTTAGAGCTAG-3'
[a]Identical to pML107 (Addgene #67639, (Laughery *et al*, 2015)) except for the sequence immediately downstream of the SNR52 promoter 5’-AGAATCGATGCATTT-3’, which was replaced with the indicated sequence. The underlined sequence is specific to the target gene.		
**Repair DNAs**		
**ID**	**Purpose**	**Sequence**
pRL1528	Repair duplex DNA for deletion of the Dsk2 STI1 domain (aa145–223)	5’-CAATCCGCTGGCCGACTTGACCAGTGCCAGATACGCTGGATATTTGAATATGCCATCTGCAGACATGTTTGGCCCGGACGGTGGTGCATTAAACAACGACGCCGGTATGGGCTCTGCAGGTGGGGCTGCCTCTGCCTTCCCCGCTCCTGGTGGCGATGCTCCAGAGGAAGGCTCCAACACGAACAC

		TACTTCCTCATCCAACACAGGGAACAACGCAGG-3’
pRL1551	Repair duplex DNA for insertion of the ALFA Tag into Dsk2 together with a silent mutation	5’-GATATGATTAGACAGAGCATGCAATTCGCAAGAATGATGGACCCTAATGCCGGTATGGGCTCTGCAGGTGGGGCTGCCTCTGCCTTCCCCGCTCCTGGTGGCGATGCTCCAGAaGAATCCAGGTTAGAGGAGGAATTGAGGAGGAGACTGACTGAGGGCTCCAACACGAACACTACTTCCTCATCCAACACAGGGAACAACGCAGGGACTAATGCAGGTA-3’
pRL1573	Repair duplex DNA for deletion of codons corresponding to Dsk2 TH2 (aa279–291) and TH3 (aa301–314)	5’-CACTACTTCCTCATCCAACACAGGGAACAACGCAGGGACTAATGCAGGTACCAACGCAGGCGCTAACACAGCTGCAAACCCAAACGCGGGAAACGCTGCATCCACCGGGCCCCCTGTACAAGCATCTCAAGCAGAAGACACCAGACCACCGGAAGAACGCTATGAACATCAATTAAGACAACTAAACGACATGGGCTTCTTCGATTTCGATAG-3’
pRL1574	Repair duplex DNA for deletion of codons corresponding to Dsk2 TH1 (aa114–134)	5’-CGTCCATCTGGTCAAGTCACAACCCAAACCACAAACCGCCAGTGCCGCTGGCGCAAATAACGCCACCGCCACCGGTGCAGCAGCTGGCACTGGCGCCACCCCGAACATGTCGTCAGGTCAAAGTGCAGGCTTCAATCCGGGCCCGGACGGTGGTGCATTAAACAACGACTCGAATAACCAAGACGAGCTGTTGAGGATGATGGAAAACCCCATCTTCCAATCGCAAATGAACGAGATGTTG-3’
pRL609	Used with pRL711 on template pYM28 to amplify a DNA fragment for insertion of eGFP behind *DSK2* at the endogenous locus	5’-GAGTAGGGTAAAAGTATATAGGTTGCGGCATCTAGACGTTATCGATGAATTCGAGCTCG-3’
pRL711	Used with pRL609 on template pYM28 to amplify a DNA fragment for insertion of eGFP behind *DSK2* at the endogenous locus	5’-CGTCCAAGGCGCTCTTGATTCACTACTGAACGGCGATGTTCGTACGCTGCAGGTCGAC-3’
pRL36	Used with pRL710 on template pBS35 to amplify a DNA fragment for insertion of mCherry behind *SCL1* at the endogenous locus	5’-GTGTTGACGCGTGTGATTTCACATTATGTTGTGGCAGGAAGATCGATGAATTCGAGCTCG-3'

pRL710	Used with pRL36 on template pBS35 to amplify a DNA fragment for insertion of mCherry behind *SCL1* at the endogenous locus	5'-TGCTGAGAACATCGAAGAAAGGCTAGTAGCAATTGCTGAACAAGATGGTCGACGGATCCCCGGG-3'
