# Supplementary material for: STI1 domain engages transient helices to mediate Dsk2 phase separation and proteasome condensation
Source: EMBO J. 2026 Feb 11;45(8):2712–38. doi: 10.1038/s44318-026-00696-1 (PMC13083955; doi:10.1038/s44318-026-00696-1)
Supplement: Supplementary file 3 — Movie EV1 [file 44318_2026_696_MOESM3_ESM.zip › Movie EV1/Legend Movie EV1.docx]

**Movie EV1:** **Simulated Dsk2 STI1 groove occupancy (UBL:UBA unbound).** Representative CALVADOS molecular dynamics simulation for full-length Dsk2 where UBL and UBA domains are left unrestrained. Colors are blue (UBL), red (STI1), purple (UBA), orange (segments corresponding to TH1, TH2, and TH3 regions). The movie frame rate corresponds to 100 ps per second.
