# Supplementary material for: STI1 domain engages transient helices to mediate Dsk2 phase separation and proteasome condensation
Source: EMBO J. 2026 Feb 11;45(8):2712–38. doi: 10.1038/s44318-026-00696-1 (PMC13083955; doi:10.1038/s44318-026-00696-1)
Supplement: Supplementary file 4 — Movie EV2 [file 44318_2026_696_MOESM4_ESM.zip › Movie EV2/Legend Movie EV2.docx]

**Movie EV2.** **Dsk2 STI1 groove occupancy time course.** Representative CALVADOS molecular dynamics simulation for full-length Dsk2 where UBL and UBA domains are restrained in the bound conformation (see Methods). Colors are blue (UBL), red (STI1), purple (UBA), orange (segments corresponding to TH1, TH2, and TH3 regions). The movie frame rate corresponds to 100 ps per second.
