# Supplementary material for: STI1 domain engages transient helices to mediate Dsk2 phase separation and proteasome condensation
Source: EMBO J. 2026 Feb 11;45(8):2712–38. doi: 10.1038/s44318-026-00696-1 (PMC13083955; doi:10.1038/s44318-026-00696-1)
Supplement: Supplementary file 5 — Expanded View Figures [file 44318_2026_696_MOESM5_ESM.pdf]

Expanded View Figures

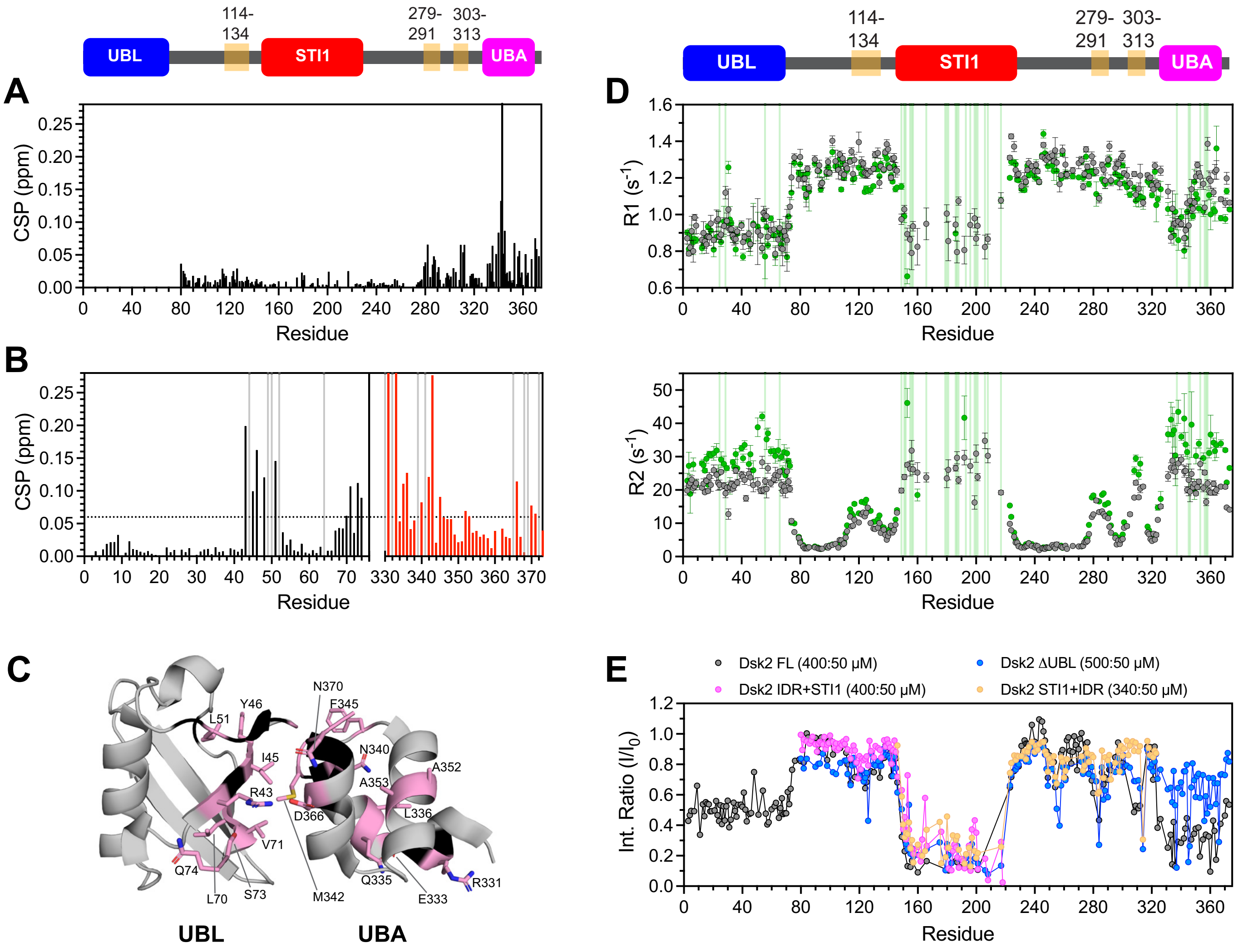

Figure EV1. Identification of UBL-UBA interactions and STI1 concentration-dependent interactions in Dsk2.

(**A**) CSPs of backbone amide resonances between Dsk2 FL and Dsk2 ΔUBL show large CSPs for residues in the UBA domain of Dsk2 indicative of UBL-UBA interactions. (**B**) CSPs are shown between Dsk2 UBL-only and Dsk2 FL (black bars), and between Dsk2 UBA-only and Dsk2 FL (red bars). Gray bars represent residues for which no amide peaks were observed (or unassigned) for Dsk2 FL. (**C**) Residues with CSPs > 0.06 ppm (above dotted black line in panel B) are highlighted as pink sticks that map to the UBL-UBA interface as shown on the crystal structure of the bound form of isolated Dsk2 UBL and UBA domains (PDB: 2BWE). (**D**) ^{15}N R_1 and R_2 relaxation rates are compared for Dsk2 at 50 μM (gray) and 400 μM (green); $n = 1$. R_1 and R_2 values are best model parameter from fit and error bars in relaxation rates are standard deviation from 500 Monte Carlo trials using RELAXFIT (see "Methods"). The concentration-dependent increase in R_2 relaxation rates (and corresponding decrease in R_1 relaxation rates) for resonances in the UBL and UBA domains suggest increased UBL-UBA intermolecular interactions with increased protein concentration. Green bars represent resonances for which there is no observable amide resonance at 400 μM; primarily affected are resonances corresponding to the STI1 domain, suggestive of intermolecular interactions involving the STI1 domain. (**E**) Concentration-dependent intensity ratios (I/I_0) of amide resonances are plotted between high (I) and low (I_0) protein concentrations of STI1 domain-containing Dsk2 variants (Appendix Table S1). Concentrations are noted in the legend above plot; intensity ratio is corrected for differences in protein concentration ("Methods"). Notably, residues within the STI1 domain of all Dsk2 constructs exhibit a similar decrease in peak intensities at higher protein concentration indicative of concentration-dependent STI1-STI1 interactions.

© The Author(s)

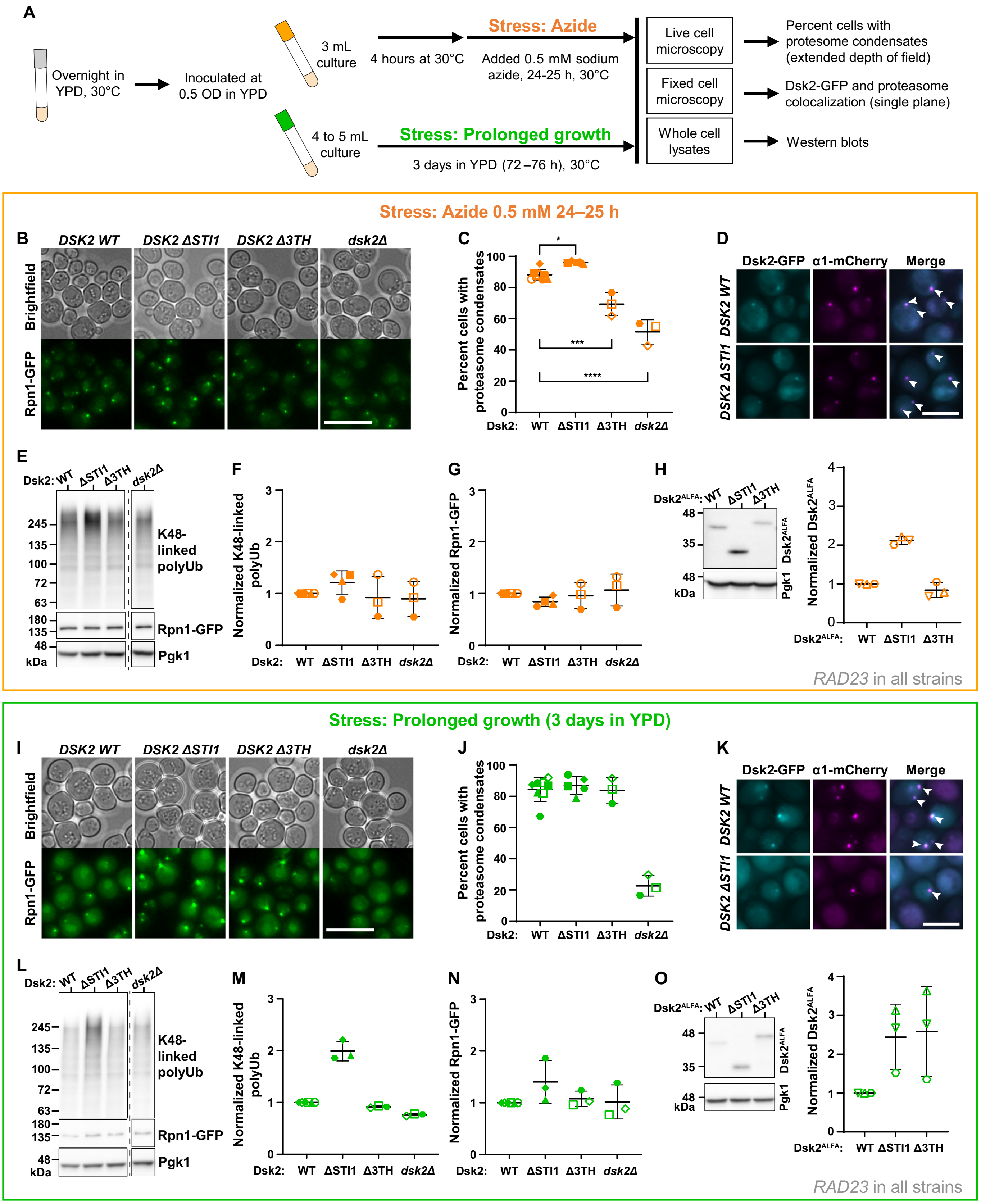
A
Overnight in YPD, 30°C
Inoculated at 0.5 OD in YPD
3 mL culture
4 hours at 30°C
Stress: Azide
Added 0.5 mM sodium azide, 24-25 h, 30°C
4 to 5 mL culture
Stress: Prolonged growth
3 days in YPD (72 –76 h), 30°C
Live cell microscopy
Fixed cell microscopy
Whole cell lysates
Percent cells with protesome condensates (extended depth of field)
Dsk2-GFP and proteasome colocalization (single plane)
Western blots
Stress: Azide 0.5 mM 24–25 h
B
DSK2 WT
DSK2 ΔSTI1
DSK2 Δ3TH
dsk2Δ
Brightfield
Rpn1-GFP
C
Percent cells with proteasome condensates
Dsk2: WT ΔSTI1 Δ3TH dsk2Δ
D
Dsk2-GFP
α1-mCherry
Merge
DSK2 WT
DSK2 ΔSTI1
E
Dsk2: WT ΔSTI1 Δ3TH dsk2Δ
K48-linked polyUb
Rpn1-GFP
Pgk1
kDa
F
Normalized K48-linked polyUb
G
Normalized Rpn1-GFP
H
Dsk2ALFA: WT ΔSTI1 Δ3TH
Dsk2ALFA
Pgk1
Normalized Dsk2ALFA
RAD23 in all strains
Stress: Prolonged growth (3 days in YPD)
I
DSK2 WT
DSK2 ΔSTI1
DSK2 Δ3TH
dsk2Δ
Brightfield
Rpn1-GFP
J
Percent cells with proteasome condensates
K
Dsk2-GFP
α1-mCherry
Merge
DSK2 WT
DSK2 ΔSTI1
L
K48-linked polyUb
Rpn1-GFP
Pgk1
M
Normalized K48-linked polyUb
N
Normalized Rpn1-GFP
O
Dsk2ALFA
Pgk1
Normalized Dsk2ALFA
RAD23 in all strains

 © The Author(s)

Figure EV2. Presence of shuttle factor Rad23 diminishes the impact of Dsk2 STI1 domain and 3TH deletions on proteasome condensate formation.

(**A**) Workflow schematic for stress-induced proteasome condensate induction in *S. cerevisiae* and cell analysis methods. (**B**–**O**) All yeast strains (in the endogenous *RAD23* background) were subjected to (**B**–**H**) ~ 24 h azide stress or (**I**–**O**) prolonged growth stress (3 days in YPD). (**B**, **I**) Representative bright-field and extended-depth-of-field epifluorescence images (GFP channel) showing proteasome puncta (endogenous Rpn1-GFP) in strains expressing Dsk2 variants after (**B**) ~ 24 h azide stress or (**I**) prolonged growth stress. Scale bar: 10 µm. (**C**, **J**) Percentage of cells with ≥1 punctum after stress. Statistics for (**C**) are $^{*}P = 0.0393$, $^{***}P = 0.0001$, $^{****}P = 0.00000003$; one-way ANOVA with Tukey's test ($\alpha = 0.05$), $n \geq 3$. (**D**, **K**) Single-plane epifluorescence images GFP-tagged endogenous Dsk2 (Dsk2-GFP: pseudocolored cyan) and mCherry-tagged α1 subunit of proteasome (α1-mCherry: pseudocolored magenta). Arrows indicate colocalization of Dsk2 with proteasome condensates in Dsk2 WT and Dsk2 ΔSTI1 cells. Scale bar: 5 µm. (**E**–**G**) Representative immunoblot of whole-cell lysates from Dsk2 variant strains after ~24 h azide stress showing (**F**) K48-linked polyubiquitin and (**G**) Rpn1-GFP levels, normalized to Pgk1 and scaled to Dsk2 WT. $n \geq 3$. (**H**) Representative immunoblot of whole-cell lysates after ~24 h azide stress (left), showing relative levels of endogenous Dsk2^{ALFA} variants normalized to Pgk1 and scaled to Dsk2^{ALFA} WT (right). $n = 3$. (**L**–**O**) Immunoblots as in (**E**–**H**) for prolonged growth stress. Each biological replicate is denoted by a single symbol wherever applicable. On plots, horizontal line and error bars represent mean and SD, respectively. Full blots in Appendix Figs. S8 and S9.

© The Author(s)

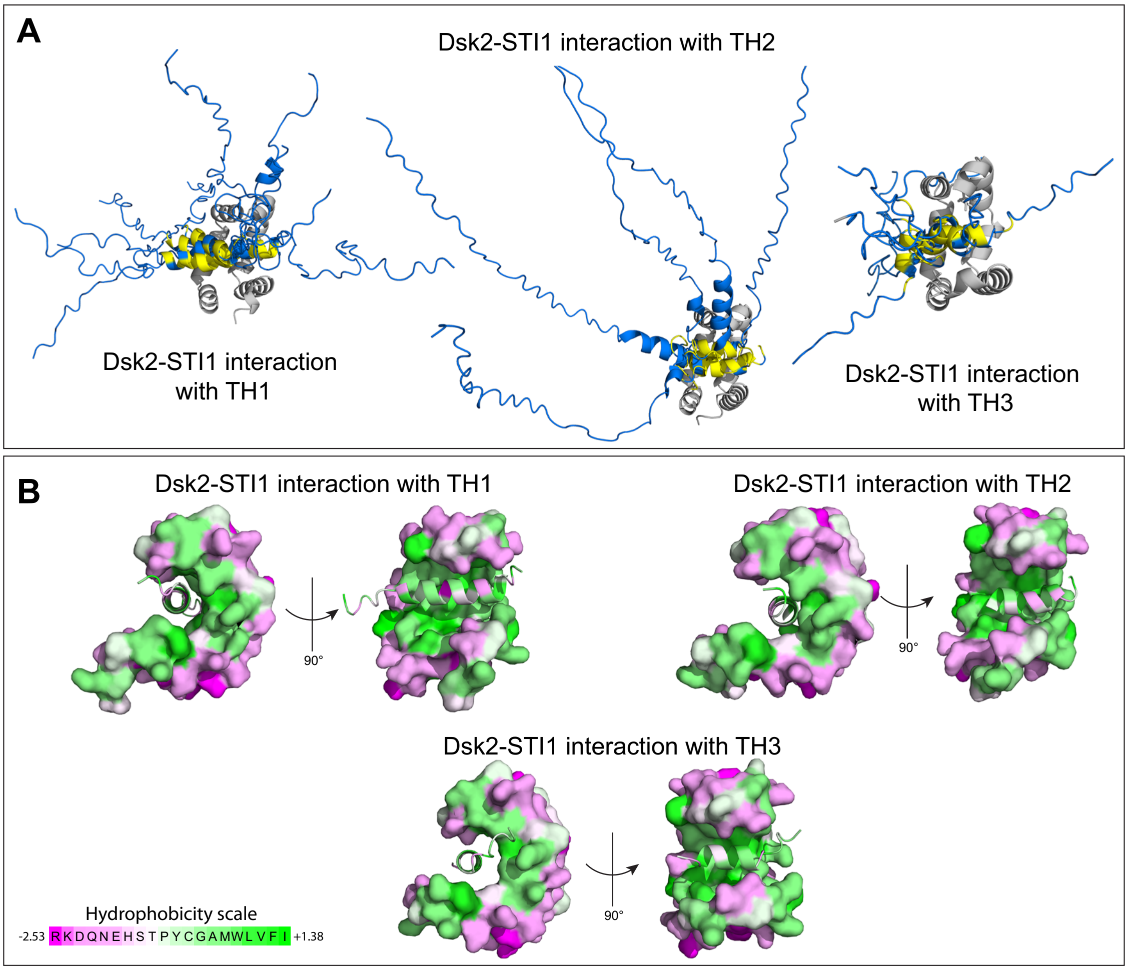

Figure EV3. Hydrophobic interactions between amphipathic helices and STI1 domain.

(**A**) Overlays of all five predicted models of AlphaFold2 multimer runs are shown for STI1-TH1, STI1-TH2, and STI1-TH3 (STI1 domain residues 147-228, TH1 region: residues 77-146, TH2 region: residues 229-291, TH3 region: residues 292-325). STI1 domain is colored gray; all TH regions (inclusive of IDRs) colored blue; TH residues with CSP > 0.04 ppm between Dsk2 FL and ΔSTI1 (from Fig. 4B) are highlighted yellow. (**B**) Hydrophobic interactions between the STI1 domain (surface representation) and transient helices TH1, TH2, and TH3 (cartoon representation) in Dsk2 using predicted AlphaFold models (same views of structures presented in Fig. 7). All amino acid residues in (**B**) are colored based on their hydrophobicity (Eisenberg et al, 1984) using Pymol.

 © The Author(s)

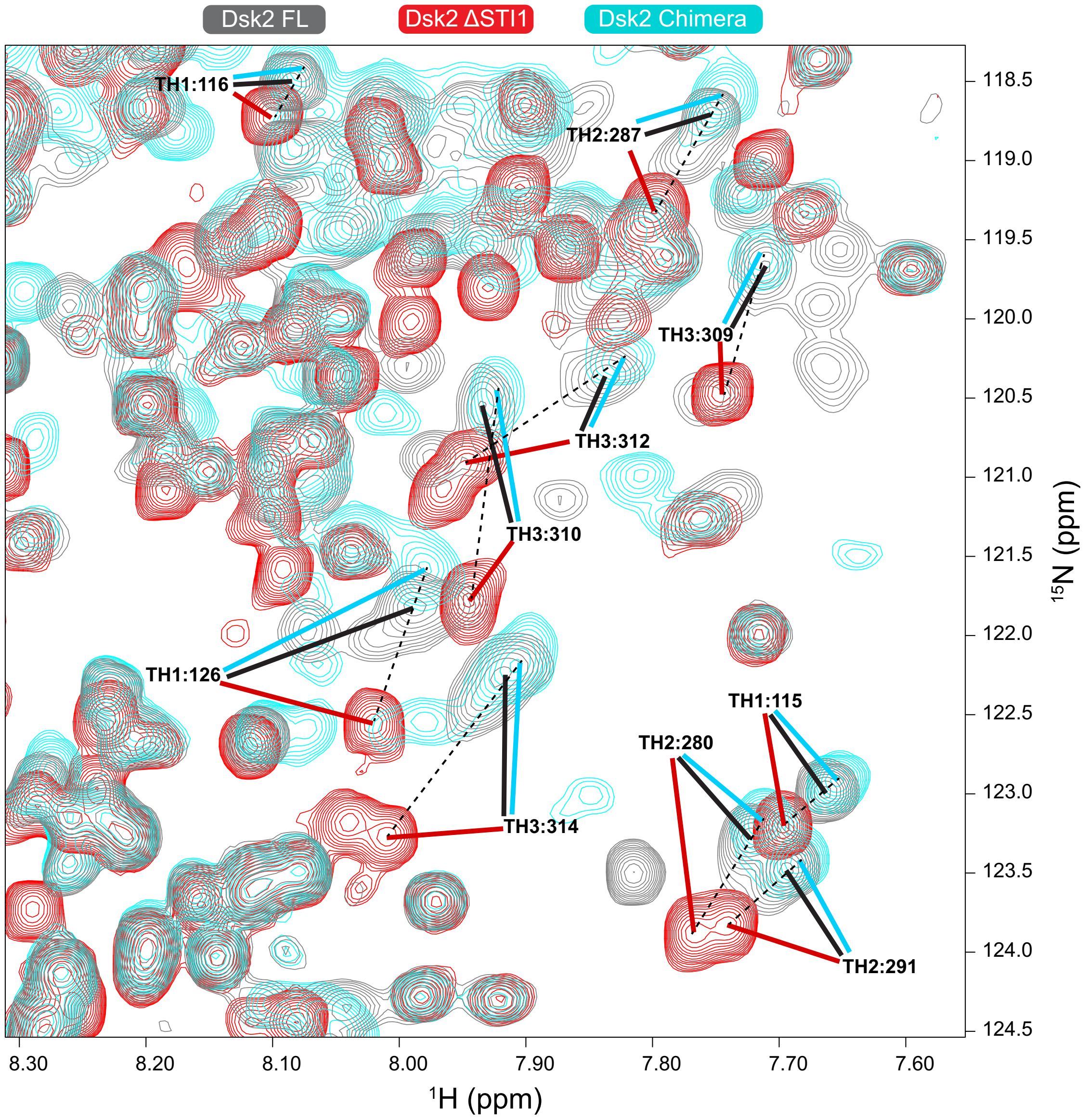

Figure EV4. Transient helices (THs) of Dsk2 interact with the UBQLN2 STI1-II domain.

Overlay of ^{1}H–^{15}N TROSY-HSQC spectra of Dsk2 FL, Dsk2 ΔSTI1, and Dsk2-Chimera (Dsk2 where STI1 is replaced with residues 379–462 (STI1-II) of UBQLN2) collected under identical conditions. Note that the backbone amide positions for Dsk2-Chimera (cyan) resonances in transient helices (annotated with "TH") nearly overlap with Dsk2 FL (gray) positions. Dotted lines are used to show trajectories of resonances across the three protein samples. The ability of UBQLN2 STI1-II domain to engage with transient helices within the Dsk2-Chimera molecule supports a general mechanism of STI1-helix interactions within UBQLNs and other STI1-containing co-chaperones.

© The Author(s)
